# Slow NMDA-Mediated Excitation Accelerates Offset-Response Latencies Generated via a Post-Inhibitory Rebound Mechanism

**DOI:** 10.1523/ENEURO.0106-19.2019

**Published:** 2019-06-17

**Authors:** Ezhilarasan Rajaram, Carina Kaltenbach, Matthew J. Fischl, Leander Mrowka, Olga Alexandrova, Benedikt Grothe, Matthias H. Hennig, Conny Kopp-Scheinpflug

**Affiliations:** 1 Division of Neurobiology, Department Biology II, Ludwig-Maximilians-University Munich, Planegg-Martinsried 82152, Germany; 2 Graduate School of Systemic Neurosciences, Ludwig-Maximilians-University Munich, Planegg-Martinsried 82152, Germany; 3 Institute for Adaptive and Neural Computation, School of Informatics, University of Edinburgh, Edinburgh EH8 9AB, United Kingdom

**Keywords:** auditory development, duration encoding, gap-detection, level-tolerance, sound-offset encoding, superior paraolivary nucleus

## Abstract

In neural circuits, action potentials (spikes) are conventionally caused by excitatory inputs whereas inhibitory inputs reduce or modulate neuronal excitability. We previously showed that neurons in the superior paraolivary nucleus (SPN) require solely synaptic inhibition to generate their hallmark offset response, a burst of spikes at the end of a sound stimulus, via a post-inhibitory rebound mechanism. In addition SPN neurons receive excitatory inputs, but their functional significance is not yet known. Here we used mice of both sexes to demonstrate that in SPN neurons, the classical roles for excitation and inhibition are switched, with inhibitory inputs driving spike firing and excitatory inputs modulating this response. Hodgkin–Huxley modeling suggests that a slow, NMDA receptor (NMDAR)-mediated excitation would accelerate the offset response. We find corroborating evidence from *in vitro* and *in vivo* recordings that lack of excitation prolonged offset-response latencies and rendered them more variable to changing sound intensity levels. Our results reveal an unsuspected function for slow excitation in improving the timing of post-inhibitory rebound firing even when the firing itself does not depend on excitation. This shows the auditory system employs highly specialized mechanisms to encode timing-sensitive features of sound offsets which are crucial for sound-duration encoding and have profound biological importance for encoding the temporal structure of speech.

## Significance Statement

Temporal features like sound-onset and sound-offset responses are crucial for acoustic pattern recognition and vocal communication. In contrast to onset responses, offset responses can be generated solely from inhibition via post-inhibitory rebounds. Here, we demonstrate that excitatory inputs are nevertheless present at offset-encoding neurons of the auditory brainstem where they serve to shorten the response latency of the offset response and stabilize the offset latency against changes in stimulus level.

## Introduction

The relative strength and temporal interaction of excitatory and inhibitory synaptic inputs determine neuronal temporal firing patterns in many parts of the brain including the auditory pathway (Wehr and Zador, 2003; Sun et al., 2010; Denève and Machens, 2016). Acoustic pattern recognition and vocal communication require precise processing of the temporal structure of sounds, such as the distinct detection of onsets and offsets, which are encoded by two dissociable channels in the auditory pathway (Anderson and Linden, 2016; Gómez-Álvarez et al., 2018; Kopp-Scheinpflug et al., 2018).

In previous studies of the central auditory system, sound-offset responses were often ignored (Kopp-Scheinpflug et al., 2018). While sound offsets are typically less abrupt (Cavaco and Lewicki, 2007) and more easily obscured by reverberation than onsets, and perceptually less prominent than onsets (Phillips et al., 2002; Deneux et al., 2016; Sohoglu and Chait, 2016), this view is changing with the discovery of neurons dedicated to detecting offsets. Recent studies suggest that neural representations of sound transients, such as offsets, are important for speech perception (Eggermont, 2015), are implicated in auditory dysfunction in brain disorders (Felix et al., 2018) and appear to be involved in short-term memory formation during auditory task performance (Elgueda et al., 2019). A previous study showed that neurons responding to sounds in a sustained fashion do not encode the end of a stimulus as reliably as those which respond specifically to the offset (Kopp-Scheinpflug et al., 2011). Detection of the end of a sound stimulus using the last sound-evoked spike in a neuron with a sustained response is highly variable, mainly due to cellular and synaptic properties changing during stimulus time. At stimulus onset, a neuron is in resting conditions and typically highly excitable, which allows fast and precise spiking. After a period of tonic activity, synaptic depression and activation of voltage-gated conductances reduce excitability, leading to less precise responses (cf. Kopp-Scheinpflug et al., 2011, their Fig. 7). Thus, a careful examination of auditory offset responses will aid in understanding their underlying coding mechanisms, both of which are prerequisites to study their behavioral relevance.

Sound-evoked offset responses have been reported in all processing stages of the ascending auditory pathway from brainstem to cortex (Kopp-Scheinpflug et al., 2018). Offset responses in higher areas such as the auditory cortex seem to be largely inherited from subcortical structures via nonoverlapping sets of excitatory inputs (Scholl et al., 2010). The de-novo generation of sound-offset responses has so far been best described for neurons in the superior olivary complex (SOC) of the mammalian auditory brainstem which exhibit acoustically-evoked offset firing at the end of a sound stimulus (Grothe, 1994; Kuwada and Batra, 1999; Behrend et al., 2002; Dehmel et al., 2002; Kulesza et al., 2003; Kulesza, 2008). These offsets can be generated via a post-inhibitory rebound mechanism that is initiated by strong glycinergic inputs and aided by the activation of hyperpolarization-activated cyclic nucleotide-modulated currents (I_H_) and T-type calcium currents (Felix et al., 2011; Kopp-Scheinpflug et al., 2011). The main source of the glycinergic input triggering these offset responses is the medial nucleus of the trapezoid body (MNTB; Grothe, 1994; Kulesza et al., 2007), which on stimulation evokes IPSCs (Kopp-Scheinpflug et al., 2011) reversing at voltages around –100 mV, well below the neurons’ resting membrane potential (Löhrke et al., 2005; Yassin et al., 2014).

It has been shown in *in vivo* measurements that this strong inhibition is essential for generating short-latency offset responses in a substantial population of SOC neurons (cat: Guinan et al., 1972; bat: Grothe, 1994; rabbit: Kuwada and Batra, 1999; gerbil: Behrend et al., 2002; Dehmel et al., 2002; rat: Kulesza et al., 2003; mouse: Kopp-Scheinpflug et al., 2011; Felix et al., 2013), but the anatomic location and number of SOC neurons with offset responses varies across mammalian species and experimental approaches. In rodents, neurons with offset responses are concentrated in the superior paraolivary nucleus (SPN) where current-clamp recordings *in vitro* show offset firing in nearly 100% of neurons (Kopp-Scheinpflug et al., 2011; Felix et al., 2013), while SPN recordings *in vivo* revealed more differential response types (Behrend et al., 2002; Dehmel et al., 2002; Kopp-Scheinpflug et al., 2011; Felix et al., 2013). These discrepancies in observing different response types between *in vivo* and *in vitro* data suggest that so far, *in vitro* experiments have missed factors that might modulate offset responses and generate more diverse response types.

Excitatory responses of SPN neurons *in vivo* have been reported either as increased sound-evoked firing rates without obvious inhibition (Behrend et al., 2002; Dehmel et al., 2002) or as responses masked by inhibition, only to be revealed after pharmacological blockade of glycinergic inputs (Kulesza et al., 2007; Jalabi et al., 2013). However, the relevance of excitatory inputs to these predominantly offset-responding neurons for auditory processing is unknown. Octopus cells of the ventral cochlear nucleus have been suggested as one source of SPN excitation based on the contralateral origin of the sound-evoked excitation, its broad frequency tuning (Dehmel et al., 2002) and anatomic tracing experiments (Thompson and Thompson, 1991; Schofield, 1995; Saldaña et al., 2009; Felix et al., 2017). An *in vitro* study also reports the presence of AMPA receptor (AMPAR)-mediated responses in the mouse SPN (Felix and Magnusson, 2016), but their function and mechanism of action are still unknown.

To gain better insight into the functional relevance of excitatory inputs during sound-offset encoding we performed immunocytochemistry, single-cell recording *in vivo*, computational modeling, and patch-clamp recordings *in vitro*. We demonstrate that the time course of slow NMDA receptor (NMDAR)-mediated excitation extends into the temporal window of post-inhibitory rebound firing. Simultaneous activation of excitation and inhibition accelerates post-inhibitory rebound responses and makes them more tolerant against changes in sound intensity *in vivo*, which is a prerequisite for sound-duration tuning and level-independent gap detection (Forrest and Green, 1987).

## Materials and Methods

All experimental procedures were reviewed and approved by the Bavarian district government (TVV AZ: 55.2-1-54-2532-38-13) and were done according to the European Communities Council Directive (2010/63/EU). C57Bl6J mice were housed in a vivarium with a normal light dark cycle (12/12 h light/dark) and food and water *ad libitum*. Mice of both sexes were used for the physiologic and anatomic experiments.

### Immunohistochemistry

Mice (P21–P35) were anesthetized with an overdose of pentobarbital and perfusion-fixed with 4% paraformaldehyde (PFA) intracardially. Following overnight postfixation in 4% PFA, coronal brainstem sections including the cochlear nucleus and the SOC of 50-µm thickness were taken using a vibrating microtome (Leica Biosystems, VT1200S). After 3 × 10-min washes in PBS, sections were transferred to a blocking solution containing 1% bovine serum albumin, 0.5% Triton X-100, and 0.1% saponin in PBS. For NMDAR staining, proteinase K treatment (1:1000 in PBS) for 20 min at 37°C was included before transferring the sections into blocking solution. Tissue was incubated for 48 h at 4°C with primary antibodies (Table 1) diluted in blocking solution. Biocytin was labeled with streptavidin conjugated to Cy3 (1:500 in blocking solution). Tissue was then washed 3 × 10 min in PBS at room temperature, before incubation for 24 h at 4°C with secondary antibodies diluted in blocking solution. Then sections were rinsed 3 × 10 min in PBS, and coverslipped with Vectashield mounting medium.

**Table 1.** Primary and secondary antibodies used for immunocytochemistry

Primary antibody	Antigene	Supplier	Catalog number	Host	Dilution
GlyT2	Synthetic from the C terminus as predicted from the cloned rat GLYT2	Millipore	AB1773	Guinea pig	1:1000
GlyT2	Recombinant protein (aa1–229 of rat GlyT2)	SySy	272003	Rabbit	1:1000
NMDA- R2c	Fusion protein from the NR2C subunit of the NMDAR	R&D Systems	PPS033	Rabbit	1:500
MAP2	Purified MAP2 isolated from bovine brain	Acris	TA336617	Chicken	1:500
VGLUT1	Purified recombinant protein of rat VGLUT 1 (aa456–560)	SySy	135304	Guinea pig	1:2000
VGLUT2	Strep-Tag fusion protein of rat VGLUT 2 (aa510–582)	SySy	135402	Rabbit	1:1000
*VGLUT3*	Peptide (C)ELNHEAFVSPRKK, corresponding to amino acid residues 533–545 of rat VGLUT3 (accession Q7TSF2); cytoplasmic, C terminus	Alomone Labs	AGC-037	Rabbit	1:300
Secondary antibody	Host species	Supplier	Catalog number	Conjugated	Dilution
Rabbit	Donkey	Dianova	711-165-152	Cy3	1:300
Rabbit	Donkey	Dianova	711-586-152	Alexa Fluor 594	1:200
Guinea pig	Donkey	Dianova	706-546-148	Alexa Fluor 488	1:200
Guinea pig	Donkey	Dianova	706-166-148	Cy3	1:300
Chicken	Donkey	Dianova	703-156-155	AMCA	1:100

### Confocal microscopy

Confocal optical sections were acquired with a confocal laser-scanning microscope equipped with HCX PL APO CS 20X/NA0.7 and HCX PL APO Lambda Blue 63×/NA1.4 immersion oil objectives (Leica). Fluorochromes were visualized with excitation wavelengths of 405 nm (emission ﬁlter 410–430 nm) for amino-methylcoumarin (AMCA), 488 nm (emission ﬁlter 510–540 nm) for Alexa Fluor 488, 561 nm (emission ﬁlter 565–585 nm) for Cy3, and 594 nm (emission ﬁlter 605–625 nm) for Alexa Fluor 594. For each optical section, the images were collected sequentially for the different fluorochromes. Stacks of 8-bit grayscale images were obtained with axial distances of 290 nm between optical sections and pixel sizes of 120–1520 nm depending on the selected zoom factor and objective. To improve the signal-to-noise ratio, images were averaged from three successive scans. RGB stacks, montages of RGB optical sections and maximum-intensity projections were assembled using the ImageJ 1.37k plugins and Adobe Photoshop 8.0.1 software.

### *In vivo* physiology

Young adult (6–16 weeks) mice of either sex (*n* = 9) were anesthetized with a subcutaneous injection of 0.01 ml/g MMF (0.5 mg/kg body weight medetomidine, 5.0 mg/kg body weight midazolam, and 0.05 mg/kg body weight fentanyl) and were placed on a temperature-controlled heating pad (WPI: ATC 1000) in a soundproof chamber (Industrial Acoustics). Depth of anesthesia was measured using the toe pinch reflex and animals responding were given supplemental MMF at 1/3 the initial dose. The mice were then stabilized in a custom stereotaxic device. An incision was made at the top of the skull, and a head post was fixed to the skull using dental cement. A craniotomy was performed above the cerebellum to access the auditory brainstem. A ground electrode was placed in the muscle at the base of the neck. Glass microelectrodes were pulled from glass capillaries so that the resistance was 5–20 MΩ when filled with 3 M KCl solution or 2 M potassium acetate with 2.5% biocytin. Signals were amplified (AM Systems, Neuroprobe 1600), filtered (300–3000 Hz; Tucker-Davis-Technologies PC1), and recorded (∼50 kHz sampling rate) with an RZ6 processor (Tucker-Davis Technologies). Python-based SPIKE software (Brandon Warren, V.M. Bloedel Hearing Research Center, University of Washington) was used to calibrate the multi-field magnetic speakers, generate stimuli and record action potentials. Stimuli consisted of pure tones (50- to 100-ms duration, 5-ms rise/fall time) at varying intensity (0- to 90-dB SPL) and were presented through hollow ear bars connected to the speakers with Tygon tubing. PSTHs were assessed at characteristic frequency (CF) and 80-dB SPL. Spike sorting and data analysis were performed offline using custom MATLAB programs. At the end of the experiment, biocytin (2.5%) was deposited at the final penetration using the current injection mode of the amplifier (+0.5 µA, 1–2 min). Thirty minutes were allowed for cellular uptake before the animal was perfused, and the tissue was processed for biocytin fluorescence as described above. Recording sites were determined using the biocytin deposition as a reference for stereotaxic reconstruction.

### *In vitro* electrophysiology

Mice of either sex P15–P22 were briefly anaesthetized with isoflurane and rapidly decapitated. Coronal brainstem sections (150–200 μm thick) containing the SOC were cut in an ice-cold high-sucrose, low-sodium artificial CSF (ACSF). Brainstem slices were maintained after slicing in normal ACSF at 37°C for 30–45 min, after which they were stored in a slice-maintenance chamber at room temperature (∼22°C). Composition of the normal ACSF: 125 mM NaCl, 2.5 mM KCl, 26 mM NaHCO_3_, 10 mM glucose, 1.25 mM NaH_2_PO_4_, 2 mM sodium pyruvate, 3 mM myo-inositol, 2 mM CaCl_2_, 1 mM MgCl_2_, and 0.5 mM ascorbic acid, pH 7.4, bubbled with 95% O_2_, 5% CO_2_. For the low-sodium ACSF CaCl_2_ and MgCl_2_ concentrations were 0.1 and 4 mM, respectively, and NaCl was replaced by 200 mM sucrose. Experiments were conducted at 36 ± 1°C, maintained by an inline feedback temperature controller and heated stage (Warner Instruments) with the recording chamber being continuously perfused with ACSF at a rate of 1–2 ml min^−1^. Whole-cell patch-clamp recordings were made from visually identified SPN neurons using an EPC10/2HEKA amplifier (HEKA Electronik), sampling at 50 kHz and filtering between 2.9 and 10 kHz. Patch pipettes were pulled from borosilicate glass capillaries (Warner Instruments) using a DMZ Universal electrode puller (Zeitz-Instuments Vertriebs GmbH), filled with a patch solution containing: 126 mM K-gluconate, 4 mM KCl, 40 mM HEPES, EGTA 5 mM HEPES, 1 mM MgCl_2_, 5 mM Na_2_phosphocreatine, 0.2% biocytin, 292 mOsm, pH was adjusted to 7.2 with KOH. For recordings of EPSCs the internal solution contained: 135 mM Cs-gluconate, 10 mM HEPES, 1 mM EGTA, 3.3 mM MgCl_2_, 3 mM Na_2_phosphocreatine, 2 mM NaATP, 20 mM TEA-Cl, 0.2% biocytin, 300 mOsm. pH was adjusted to 7.2 with CsOH. Data were corrected for liquid junction potentials of –13.8 and –13.7 mV for the potassium-based and the cesium-based internal solutions, respectively. Electrode resistance was between 2.4 and 6 MΩ. Synaptic responses were evoked by afferent fiber stimulation with either concentric or bipolar (FHC) electrodes. Voltage pulses were generated by the HEKA amplifier and post-amplified by an isolated pulse stimulator (AM Systems). Synaptic conductances were calculated from the synaptic currents: G = PSC/(V_m_ – E_PSC_), with PSC being the postsynaptic current, V_m_ being the holding potential (–60 mV for inhibition; 40 mV for excitation), E_PSC_ being the reversal potential of the postsynaptic current (E_EPSC_: 0 mV; E_IPSC_: –100 mV).

### Computational model

Two simple, single-compartment models of a SPN neuron were simulated using NEURON (version 7.5; Hines and Carnevale, 2001). In both models, the basic set-up of the neuron, including the membrane properties and the ionic channels, is identical to the model developed by Kopp-Scheinpflug et al. (2011), available on ModelDB (https://senselab.med.yale.edu/modeldb/ShowModel.asp?model=139657), accession number 139657. In the two current models, the excitatory noise was removed, and four excitatory synapses were added in addition to the inhibitory synapses already present. The synapses are modeled using the NEURON built-in function Exp2Syn. In both models, the excitatory and inhibitory stimuli consist of 10 such spikes with 10-ms gaps, resulting in a total stimulus duration of 100 ms. The excitatory and inhibitory conductances and the respective time constants used in the model are provided by the patch-clamp experiments performed for this paper. In the first model (including AMPA currents), all four excitatory synapses are simulating AMPA synapses, with a reversal potential E_rev_AMPA_ = 0 mV, a rise time constant of τ_1_ = 0.1 ms and a decay time constant of τ_2_ = 0.9 ms. In the second model (including AMPA and NMDA currents), only two excitatory synapses are simulating AMPA synapses, with the same parameters as in the first model; while the two other excitatory synapses are simulating simplified NMDA synapses, with reversal potential E_rev_NMDA_ = 0 mV, a rise time constant of τ_1_ = 3 ms, and a decay time constant of τ_2_ = 9 ms. The conductances of the excitatory synapses were varied in both models. Both models included 14 inhibitory synapses with reversal potential E_rev_inh_ = –100 mV, a rise time constant τ_1_ = 0.1 ms, a decay time constant τ_2_ = 2 ms and a peak conductance of 82 ns and 41 nS. A simplified depression of the input synapses was modeled using the steady-state depression values collected for this paper.

### Experimental design and statistical analysis

In the text data are presented in parenthesis as (median; 25/75 quartiles or as mean ± SEM; test: *p* value) unless indicated otherwise. In the figures, data are presented as medians (lines in boxes); 25/75 quartiles (boxes); and 10th/90th percentiles (whiskers) in addition to individual data points. Statistical analyses of the data were performed with SigmaStat/SigmaPlot. Normality was tested by the Shapiro–Wilk test. Comparisons between different data sets were made depending on the distribution of the data using parametric tests for normally distributed data (two-tailed Student’s *t* test for comparing two groups and ANOVA for comparing three or more groups). When the normality assumption was violated, nonparametric tests (Mann–Whitney rank-sum test for comparing two groups and Kruskal–Wallis ANOVA on ranks for comparing three or more groups) were used. Paired *t* tests or Wilcoxon signed rank tests were used when two data sets were recorded from individual neurons under different conditions. Differences were considered statistically significant at *p* ≤ 0.05 and presented in the figures as n.s. for nonsignificant differences and as *p ≤ 0.05, **p ≤ 0.01, and ***p ≤ 0.001 for significant differences. Intrinsic properties as well as postsynaptic current amplitudes and kinetics were analyzed using Stimfit software (Guzman et al., 2014). For data acquired with *in vivo* single-unit recording or patch-clamp recording; *n* is the number of neurons, with two to three brain slices per animal and at least three animals per group.

## Results

### SPN neurons receive glycinergic and glutamatergic synaptic input

**Figure 1.**
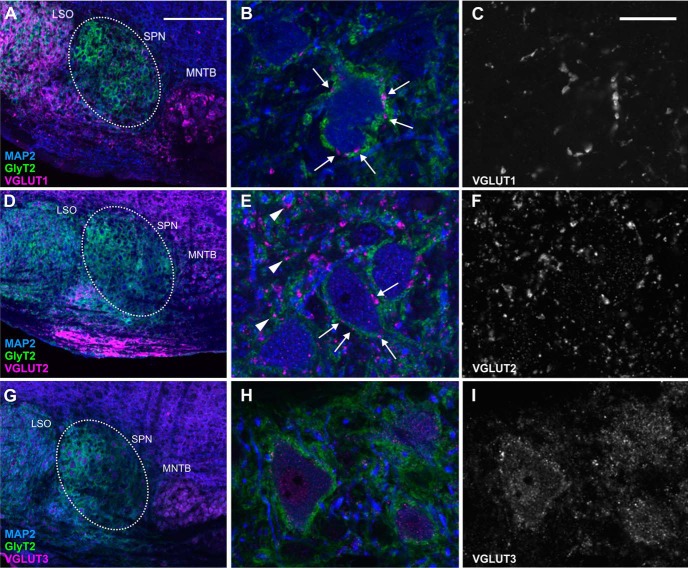
Histochemical profile of excitatory and inhibitory inputs to the SPN. ***A***, ***B***, ***D***, ***E***, ***G***, ***H***, Glycinergic input forms the most prominent input to SPN (white dotted circle in ***A***, ***D***, ***G***) and is depicted by neuronal GlyT2 (green) labeling. Immunolabeling for microtubule associated protein 2 (MAP2, blue) is used as a neuronal marker in all images. Glutamatergic inputs are shown by labeling for the VGLUTs (magenta): VGLUT1 (***A***–***C***), VGLUT2 (***D***–***F***), VGLUT3 (***G***–***I***). ***A–F***, While both VGLUT1 (***B***, white arrows) and VGLUT2 (***E***, white arrows) positive synaptic boutons are present at the soma, VGLUT2 boutons are also seen in the neuropil (***E***, white arrowheads). ***G–I***, VGLUT3 shows only weak somatic but no presynaptic bouton labeling. Scale bars = 200 µm (left images) and 20 µm (middle and right images).

We previously showed that only inhibitory synaptic input is required to generate offset responses in the SPN (Kopp-Scheinpflug et al., 2011). Here, this was corroborated by immunocytochemistry showing strong expression of the neuronal glycine transporter type 2 (GlyT2), which labels glycinergic synaptic terminals around the soma of SPN neurons (Fig. 1, green boutons). However, the additional presence of excitatory synapses was confirmed by labeling for the vesicular glutamate transporter (VGLUT) types 1–3 (Fig. 1*A*,D,*G*). While VGLUT1 and VGLUT2 positively labeled presynaptic terminals in SPN (Fig. 1*B*,*C*,*E*,*F*), the signal strength for VGLUT3 was quite low and somatic rather than in the presynaptic boutons (Fig. 1*H*,*I*).

### SPN neurons with sound-offset responses can exhibit moderate excitatory responses during contralateral sound stimulation

**Figure 2.**
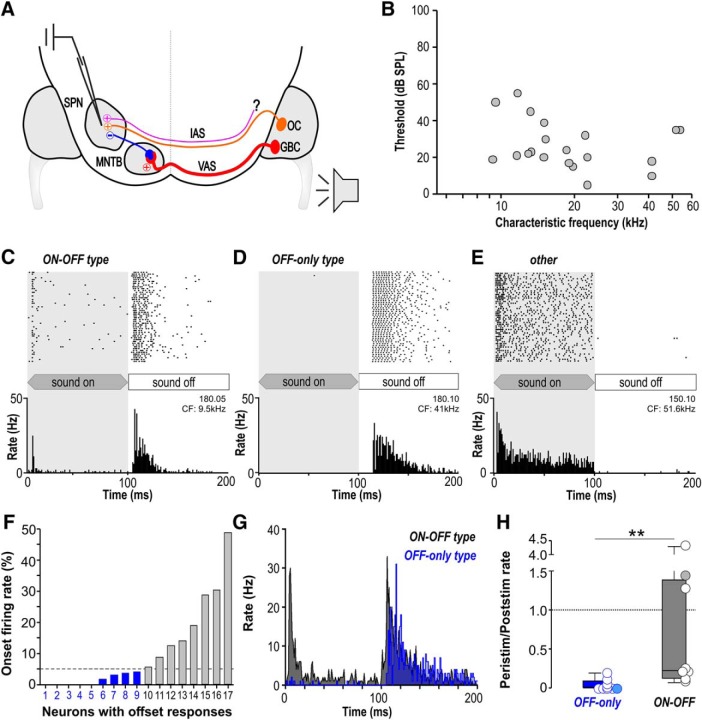
SPN neurons with post-inhibitory rebound responses at sound offset can also show excitatory responses during sound. ***A***, Schematic of a sound-offset encoding circuit in the mammalian brainstem. Globular bushy cell (GBC) axons project via the ventral acoustic stria (VAS) to the contralateral MNTB to form one-to-one connections via the giant calyx of Held synapses. MNTB neurons then project to neurons of the SPN. SPN cells also receive excitatory input from octopus cells (OCs) and possibly other, yet unknown sources (?) in the contralateral ventral cochlear nucleus via the IAS. ***B***, Plot of CFs and auditory thresholds of all SPN neurons recorded in this sample. ***C–E***, Raster plots and PSTHs (at CF, 80-dB SPL, 50 trials) shown for three individual SPN neurons with different response patterns. ***C***, ON-OFF type, showing a peristimulus response related to sound onset followed by a response related to sound offset. ***D***, OFF-only type, showing responses related to sound offset, but no peristimulus firing. ***E***, Response type showing only peristimulus firing but no poststimulus response at sound offset. ***F***, Distribution of neurons with offset responses depending on how much of their overall firing activity during the combined 200-ms peristimulus and poststimulus period was present within the first 20 ms of the response. The dashed line represents the 5% criterion we used to classify the neurons into either OFF-only type neurons (blue: 1–9) or ON-OFF type neurons (gray: 10–17). ***G***, Average temporal response patterns for OFF-only type neurons (blue) and ON-OFF type neurons (gray). ***H***, Peristimulus-to-poststimulus ratio for OFF-only type neurons (blue) and ON-OFF type neurons (gray). Firing rates were averaged over the whole peristimulus time window and divided by the average of the whole poststimulus time window. Equal firing rates in both time windows result in a ratio of zero (dotted line). Filled circles represent the ratios for the example cells shown in ***C***, ***D***. Note that OFF-only neurons with ratios larger than zero exhibit spontaneous APs which also appear during the peristimulus time window but not concentrated within the first 20 ms to form an onset response. **p ≤ 0.01.

To explore the contribution of excitatory inputs to signal processing in neurons with sound-offset responses, spikes were recorded from single SPN neurons in anesthetized mice *in vivo*, during (peristimulus) and after (poststimulus) the presentation of sound (Fig. 2*A*). The sample of 20 SPN neurons had CFs ranging from 9.25 to 50.4 kHz (Fig. 2*B*); 85% (17/20) of these neurons showed a burst of increased firing at the end of contralateral sound stimulation (offset responses) and little or no firing during sound presentation (Fig. 2*C*,*D*), consistent with a prevalent inhibitory input and the dominance of offset responses reported in previous studies (Dehmel et al., 2002; Kulesza et al., 2003; Felix et al., 2013). Only 3/20 SPN neurons did not exhibit offset firing, but responded with an onset, primary-like or sustained firing pattern during sound stimulation (Fig. 2*E*). These neurons that fired spikes only during but not after the stimulus were not included in further analyses. Of SPN neurons with offset responses, 53% (9/17) additionally showed excitatory responses during sound stimulation that exceeded 5% of the respective neurons’ overall firing rate (Fig. 2*F*). These neurons will be further referred to as ON-OFF type neurons (Fig. 2*F*,*G*, gray) and will be contrasted against neurons that exhibit offset responses without peristimulus excitation (OFF-only type; Fig. 2*F*, blue). Average temporal response patterns show ON-OFF type neurons with either an onset or a primary-like temporal response pattern during sound followed by a poststimulus offset response (Fig. 2*G*, gray histogram). Onset or primary-like temporal response patterns are associated with octopus cells in the ventral cochlear nucleus (Rhode et al., 1983) which are one source of excitatory input to the SPN (Thompson and Thompson, 1991; Schofield, 1995; Saldaña et al., 2009; Felix et al., 2017). The average response of the OFF-only type neurons is characterized by only little spontaneous firing during sound followed by a slightly delayed poststimulus offset response (Fig. 2*G*, blue histogram). The ratio of peristimulus to poststimulus rate is significantly larger in the ON-OFF type neurons (ON-OFF type: 0.22; 0.12/1.39; *n* = 9; OFF-only type: 0; 0/0.09; *n* = 8; Mann–Whitney rank-sum test: *p* = 0.002; Fig. 2*H*).

### SPN neurons that exhibit additional excitation have accelerated and level-independent offset-response latencies

**Figure 3.**
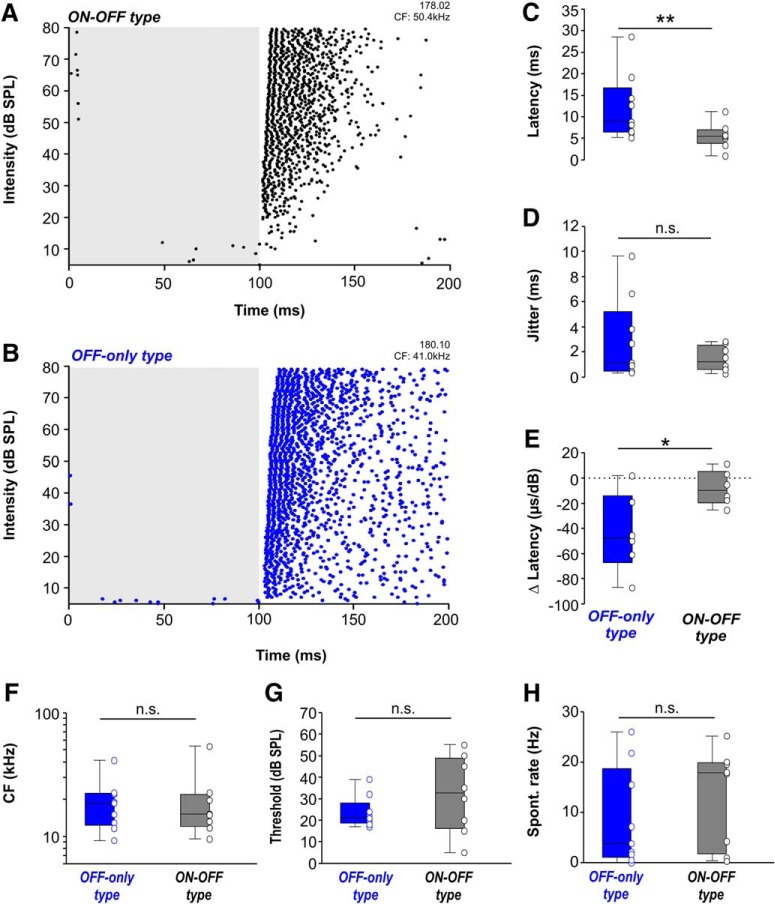
ON-OFF type SPN neurons have shorter offset-response latencies and less level dependence than OFF-only type neurons. ***A***, ***B***, Raster plot at CF and changing intensity levels for representative (***A***) ON-OFF type and (***B***) OFF-only type neurons. Each dot represents an action potential. Gray-shaded area indicates sound duration. ***C***, ***D***, Distribution of (***C***) offset latencies and (***D***) jitter for OFF-only type (blue) and ON-OFF type (gray) neurons at CF/80-dB SPL. Statistical assessment in ***C***, ***D*** was unaltered if the two extreme values were removed. ***E***, Average change of offset-response latency per dB intensity change for OFF-only type (blue) and ON-OFF type (gray) SPN neurons. OFF-only type neurons (blue) showed a significantly larger variability of offset-response latency with changes in intensity. Latency change/dB was not significantly different from zero (dotted line) for ON-OFF type neurons (=level invariant). ***F–H***, Between SPN neurons of either OFF-only type or ON-OFF type, physiologic parameters like (***F***) CFs, (***G***) thresholds, and (***H***) spontaneous firing rates are not significantly different. *p ≤ 0.05, **p ≤ 0.01, n.s. = non-significant.

The average temporal response pattern in Figure 2*G* suggests that offset-response latencies are shorter in ON-OFF type neurons compared to OFF-only type neurons. To investigate potential differences in latency in more detail, offset responses were recorded at CF for increasing sound intensities (Fig. 3*A*,*B*). Indeed, offset-response latencies were significantly faster for ON-OFF type neurons (5.55 ms, 3.80/6.97 ms, *n* = 8) than for OFF-only type neurons (8.84 ms, 6.38/16.75 ms, *n* = 9; Mann–Whitney rank-sum test: *p* = 0.018; Fig. 3*C*). However, no difference was observed for the variability of the offset-response latency (jitter) between *ON-OFF* and OFF-only type neurons (jitter_ON-OFF_: 1.23 ms; 0.62/2.54 ms, *n* = 8; jitter_OFF-only_: 1.11 ms; 0.45/5.23 ms; *n* = 9; Mann–Whitney rank-sum test: *p* = 0.665; Fig. 3*D*), suggesting that the intrinsic properties of the cells that regulate precise spike firing are not the reason for the difference in latencies.

A common feature in stimulus encoding across different sensory modalities is that response latencies decrease with increasing stimulus intensity (auditory: Kitzes et al., 1978; Klug et al., 2000; somatosensory: Mountcastle et al., 1957; visual: Morgan and Thompson, 1975). In contrast, offset-response latencies of both ON-OFF type (Fig. 3*A*) and OFF-only type (Fig. 3*B*) SPN neurons did not show a strong dependency on stimulus intensity but remained rather constant over a large sound intensity range or even showed a trend of increasing latencies with increasing intensities (single cell examples shown in Fig. 3*A*,*B*). On average, there were no significant changes in latency per decibel sound intensity for the ON-OFF type neurons (–9.83 µs/dB; –19.46/5.11 µs/dB; *n* = 6; Fig. 3*E*), while changes in latencies per decibel for the OFF-only type neurons were significantly larger (–47.79 µs/dB; –67.37/–13.87 µs/dB; *n* = 6, Kruskal–Wallis ANOVA on ranks followed by Dunn’s multiple comparisons versus a zero change control: *p* = 0.032).

If the neurons firing either ON-OFF or OFF-only responses represent two distinct populations, they may inhabit different locations in the SPN. This was tested by testing whether ON-OFF or OFF-only responding neurons have similar or different CFs. The CFs of the offset response for ON-OFF type neurons (15.1 kHz; 12.0/21.8 kHz; *n* = 8) were compared with those for OFF-only type neurons (18.5 kHz; 12.3/22.3 kHz; *n* = 9) but no significant difference was found (Mann–Whitney rank-sum test: *p* = 1.000; Fig. 3*F*). Tonotopically organized glycinergic projections from MNTB into SPN suggest that neurons tuned to high frequencies are located more medially and neurons tuned to low frequencies are located more laterally (Banks and Smith, 1992). As a result, neurons with different CFs should inhabit separate anatomic locations within the tonotopic axis of the SPN, which was not observed in the present sample of ON-OFF and OFF-only type neurons and suggests, that neurons having ON-OFF or OFF-only responses are part of a continuum with the only difference being differently balanced excitation and inhibition. Response thresholds of offset responses were also not significantly different between ON-OFF type (32-dB SPL; 16.3/48.8-dB SPL; *n* = 8) and OFF-only type neurons (21-dB SPL; 18.5/28-dB SPL; *n* = 9; Mann–Whitney rank-sum test: *p* = 0.386; Fig. 3*G*).

The occurrence of excitatory responses in some but not all neurons of a nucleus with generally dominant inhibition could also be attributed to a reduced inhibitory constraint in ON-OFF type neurons, resulting in higher spontaneous firing rates compared to OFF-only type neurons. Inhibition in SPN neurons is provided by strong glycinergic input from MNTB neurons. Despite high firing rates during sound stimulation, MNTB neurons are spontaneously active with an average rate of 20–30 Hz (Kopp-Scheinpflug et al., 2008) which tonically suppresses SPN activity. Spontaneous firing rates in SPN were not significantly different between ON-OFF type neurons (13.25 ± 3.5 Hz) compared to OFF-only type neurons (8.73 ± 3.7 Hz; two-tailed *t* test: *p* = 0.359; *t* = 0.946; df = 15; Fig. 3*H*), implying a similar strength of inhibitory innervation across neurons. Across the population of cells tested, ON-OFF type and OFF-only type neurons do not exhibit differences in location or general physiologic properties suggesting them to belong to one cell population with differing strength of excitatory input.

### Offset-response thresholds are more sensitive than peristimulus-response thresholds

**Figure 4.**
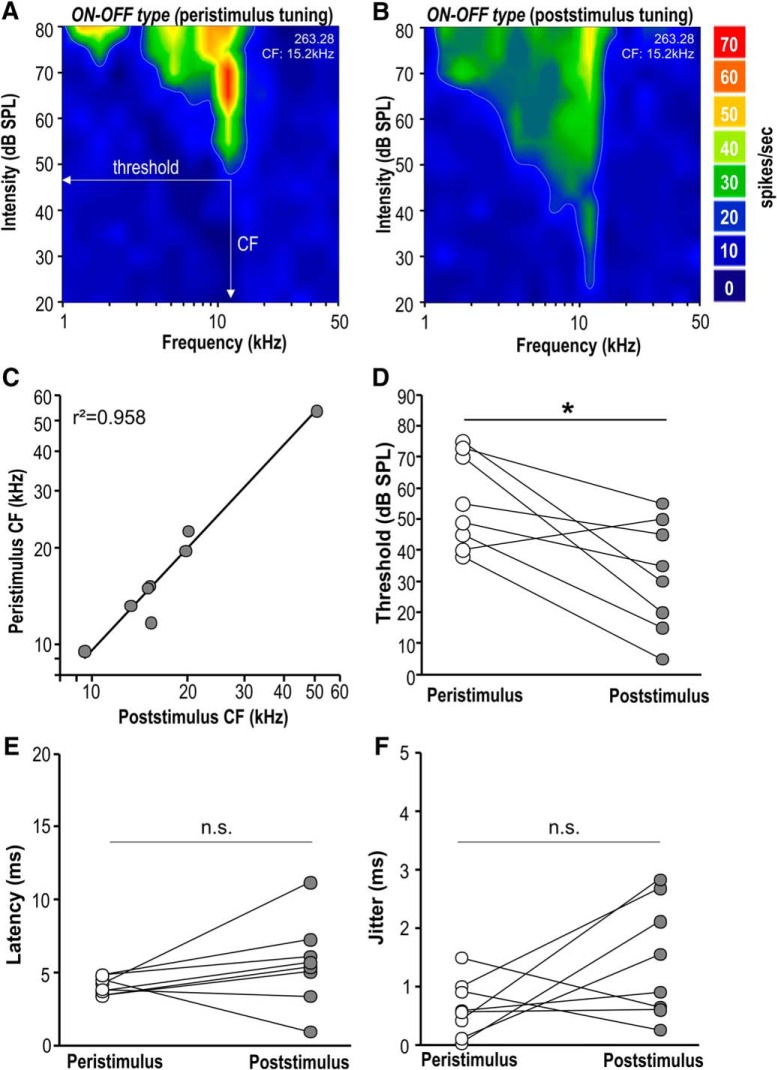
Within ON-OFF type SPN neurons, peristimulus responses have similar tuning, but elevated threshold compared to their poststimulus offset-responses. ***A***, ***B***, Frequency-intensity response maps for an individual ON-OFF type SPN neuron plotted for the peristimulus response (***A***) and for the poststimulus response after sound cessation (***B***). ***C***, Linear correlation between peristimulus and poststimulus CFs for individual ON-OFF type SPN neurons. ***D***, Thresholds for the responses during sound (peristimulus, white circles) are higher than thresholds for responses after sound termination (poststimulus, gray circles). ***E***, ***F***, Latencies and jitter were not significantly different between peristimulus and poststimulus response of individual ON-OFF type SPN neurons. *p ≤ 0.05, n.s. = non-significant.

Comparing the peristimulus and poststimulus excitatory responses within each ON-OFF type SPN neuron revealed that their spectral tuning largely overlapped (Fig. 4*A*,*B*), which is in contrast to results from auditory cortex (Scholl et al., 2010; Sollini et al., 2018) and will be discussed later. There was no significant difference between the peristimulus and poststimulus CFs for ON-OFF type neurons (CF_peristim_: 19.8 ± 4.5 kHz; *n* = 8; CF_poststim_: 20.0 ± 4.9 kHz; *n* = 8; two-tailed paired *t* test: *p* = 0.74; *t* = –0.345; df = 7; Fig. 4*C*). Thresholds were significantly lower for poststimulus offset responses (32 ± 6-dB SPL) compared to excitatory peristimulus responses (56 ± 5-dB SPL; *n* = 8; two-tailed paired *t* test: *p* = 0.011; *t* = 3.407; df = 7; Fig. 4*D*). For each ON-OFF type neuron first-spike latencies for the excitatory peristimulus response (4.12 ± 0.2 ms) were barely faster than those of the offset response following sound cessation (5.64 ± 1.0 ms; two-tailed paired *t* test: *p* = 0.188; *t* = 1.458; df = 7; Fig. 4*E*), indicating a high speed in generating offset responses despite the additional synaptic delay arising during the sign conversion in the MNTB. Jitter as a measure of the temporal precision of the first spike in the response was also not significantly different between the peristimulus (0.64 ± 0.17 ms) and the poststimulus (1.45 ± 0.35 ms) response (two-tailed paired *t* test: *p* = 0.111; *t* = 1.822; df = 7; Fig. 4*F*). The mean latency of ∼4 ms for the peristimulus response is similar to that of other SOC neurons in mouse (Kopp-Scheinpflug et al., 2008). Although the average temporal response pattern of the ON-OFF type neurons shows a primary-like pattern (Fig. 2*G*), 5/8 ON-OFF type neurons show onset firing patterns. To investigate whether primary-like or onset-responses can influence the offset response, *in vivo* recordings of spikes do not provide sufficient information about possible subthreshold activity that lasts throughout the stimulation, which will more appropriately be measured by whole-cell patch-clamp recordings *in vitro*.

### Strength of inhibition outweighs excitation in SPN neurons

**Figure 5.**
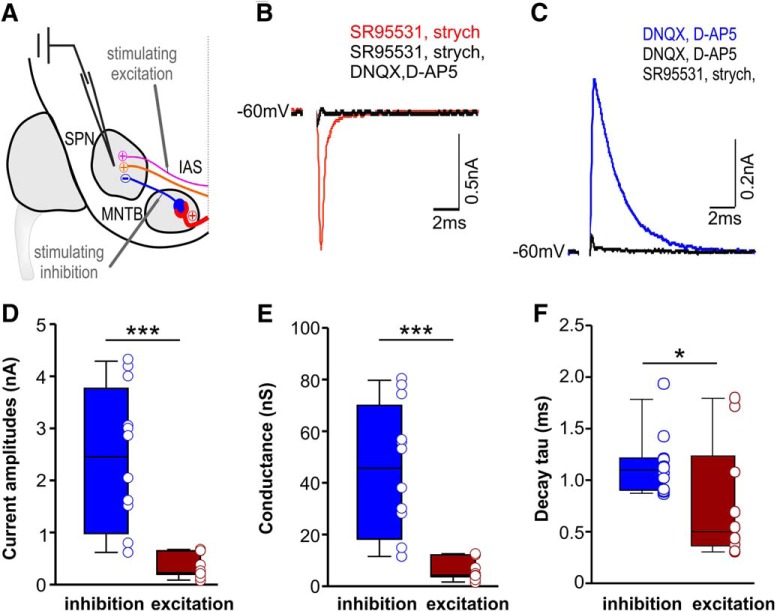
Comparison of synaptic strength between EPSCs and IPSCs in SPN neurons. ***A***, Schematic of the sound-offset encoding circuit depicting the positions of the stimulating electrodes for eliciting either excitation or inhibition. ***B***, ***C***, Voltage-clamp traces of pharmacologically isolated EPSCs (***B***) evoked by stimulating IAS and IPSCs (***C***) evoked by stimulating MNTB (average of 10 traces). Black traces indicate the blockade of (***B***) EPSCs or (***C***) IPSCs. ***D***, Average IPSC (blue) and EPSC (red) amplitudes measured in response to maximum stimulation. ***E***, EPSCs and IPSCs expressed as conductance reveals that mean IPSG values are more than five times larger than EPSGs. ***F***, Average decay time constants (τ) of EPSCs and IPSCs. Tau_IPSC_ is significantly slower than Tau_EPSC_. ***p ≤ 0.001.

Whole-cell patch-clamp recordings *in vitro* were made of SPN neurons. Neurons had resting membrane potentials of –61.74 ± 0.79 mV, an average input resistance of 74.39 ± 10.94 MΩ and an average membrane capacitance of 65.63 ± 7.78 pF (*n* = 23). Glutamatergic EPSCs were regularly activated in neurons throughout the SPN. For electrical stimulation, a concentric stimulating electrode was placed on the intermediate acoustic stria (IAS) medial to the SPN and just dorsal to the MNTB (Fig. 5*A*). EPSCs were pharmacologically isolated by adding the GABA_A_ receptor blocker SR95531 (20 µM) and the glycine receptor blocker strychnine (1 µM) to the bath solution (Fig. 5*B*). Glycinergic IPSCs were evoked by direct electrical stimulation of the ipsilateral MNTB using a concentric stimulating electrode and were pharmacologically isolated by adding the AMPAR blocker 6,7-dinitroquinoxaline-2,3-dione (DNQX; 10 µM) and the NMDAR blocker D-2-amino-5-phosphonopentanoic acid (D-AP5; 50 µM) to the bath solution (Fig. 5*C*). At physiologic holding voltages near the neurons resting membrane potential (–60 mV), IPSC amplitudes were significantly larger than EPSCs (IPSC: 2.46; 0.98/3.77; *n* = 12; EPSC: 0.23; 0.20/0.65; *n* = 11; Mann–Whitney rank-sum test: *p* ≤ 0.001; Fig. 5*D*). The considerable difference between the strength of excitation and that of inhibition was even more obvious when both were expressed as conductances (IPSG: 45.65; 18.22/70.02; *n* = 12; EPSG: 4.26; 3.72/12.08; *n* = 11; Mann–Whitney rank-sum test: *p* ≤ 0.001; Fig. 5*E*). The decay time constants of IPSCs were significantly slower than that of EPSCs (Tau_IPSC_: 1.09; 0.90/1.21; *n* = 12; Tau_EPSC_: 0.49; 0.36/1.24; *n* = 11; Mann–Whitney rank-sum test: *p* = 0.038; Fig. 5*F*).

### NMDAR-mediated currents are present in SPN neurons

**Figure 6.**
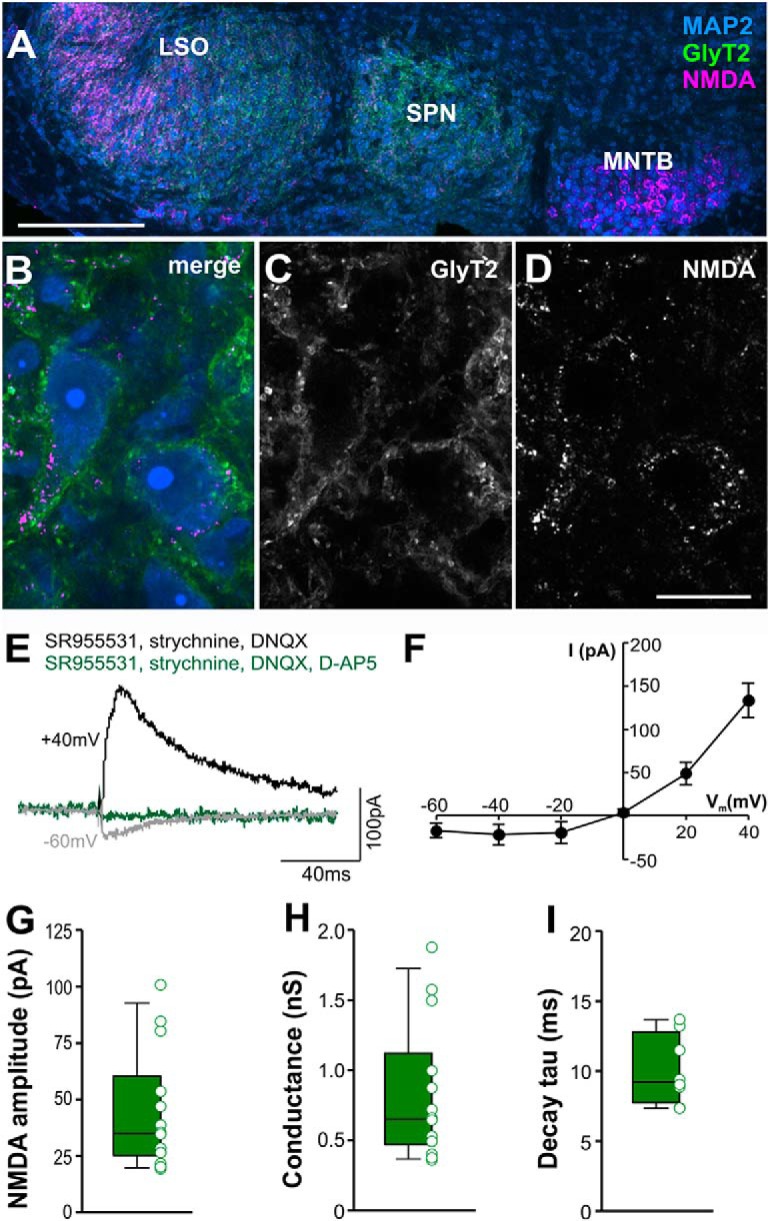
SPN neurons express NMDARs which mediate moderate EPSCs at depolarized voltages. ***A***, Low-power image of SOC showing NMDA immunoreactivity (magenta), which was strong in the MNTB and the lateral superior olive (LSO), and to a lesser degree present in the SPN. GlyT2 labeling (green) identifies the outline of the SPN. MAP2 (blue) was used as a neuronal marker. ***B–D***, Higher magnification images show that NMDARs are present in SPN neurons. ***E***, Voltage-clamp traces of pharmacologically isolated NMDA currents at –60 mV (gray) and at +40 mV (black). Currents were blocked by D-AP5 (green). ***F***, Average NMDA currents show the typical nonlinearity due to the Mg^2+^ block at hyperpolarized membrane voltages. ***G***, Amplitude of NMDA currents at +40 mV. ***H***, NMDA conductance. ***I***, Decay time constants (τ) of NMDA currents at +40 mV. Scale bars = 200 µm (***A***) and 20 µm (***B–D***).

Glutamate released from excitatory synaptic inputs typically activates AMPAR and/or NMDAR with fast and slow kinetics, respectively. Current-clamp recordings of SPN neurons near the neuronal resting potential suggested that excitatory responses are primarily mediated by AMPARs (Felix and Magnusson, 2016). Here, we used immunocytochemistry to probe for the presence of NMDARs and performed voltage-clamp recordings to assess the strength of NMDAR-mediated currents at different membrane potentials. NMDAR expression is evident in mature SPN neurons, although weaker compared to neurons in the MNTB or LSO (Fig. 6*A–D*). Electrophysiological measurement of NMDA currents in whole-cell voltage-clamp mode revealed the characteristic voltage dependence causing larger currents once the membrane voltage reaches depolarized values (Fig. 6*E*,*F*) and showed that they were sensitive to the NMDAR antagonist D-AP5 (Fig. 6*E*). NMDA currents in SPN neurons (35.0pA; 25.2/60.3pA; *n* = 14; Fig. 6*G*) were smaller than in MNTB (Steinert et al., 2010), but similar to LSO (Alamilla and Gillespie, 2011; Pilati et al., 2016) and MSO (Smith et al., 2000; Couchman et al., 2012). NMDA conductances calculated from these currents ranged from 0.36 to 1.88 nS (0.65; 0.47/1.12 nS; *n* = 14). The small, yet prevalent NMDA currents resemble the general decline of NMDA currents in the auditory brainstem following hearing onset. However, as Steinert and colleagues have shown for neurons in the neighboring MNTB, after the initial reduction in amplitudes, NMDA currents reach a steady state with no signs of further decline after about two weeks of age (Steinert et al., 2010), corroborating the presence of NMDA current in the SPN for ages of 15 d and older. Decay time constants of the NMDAR-mediated EPSCs ranged from 7.34 to 13.69 ms (9.21 ms; 7.74/12.79 ms; *n* = 8; Fig. 5*H*). In later experiments AMPAR and NMDAR responses will be activated and blocked in unison by a cocktail of DNQX/D-AP5 and together will be contrasted against glycinergic inhibition to reveal the contribution of excitation to ON-OFF type SPN neurons.

### Differential short-term plasticity between excitatory and inhibitory SPN synapses

**Figure 7.**
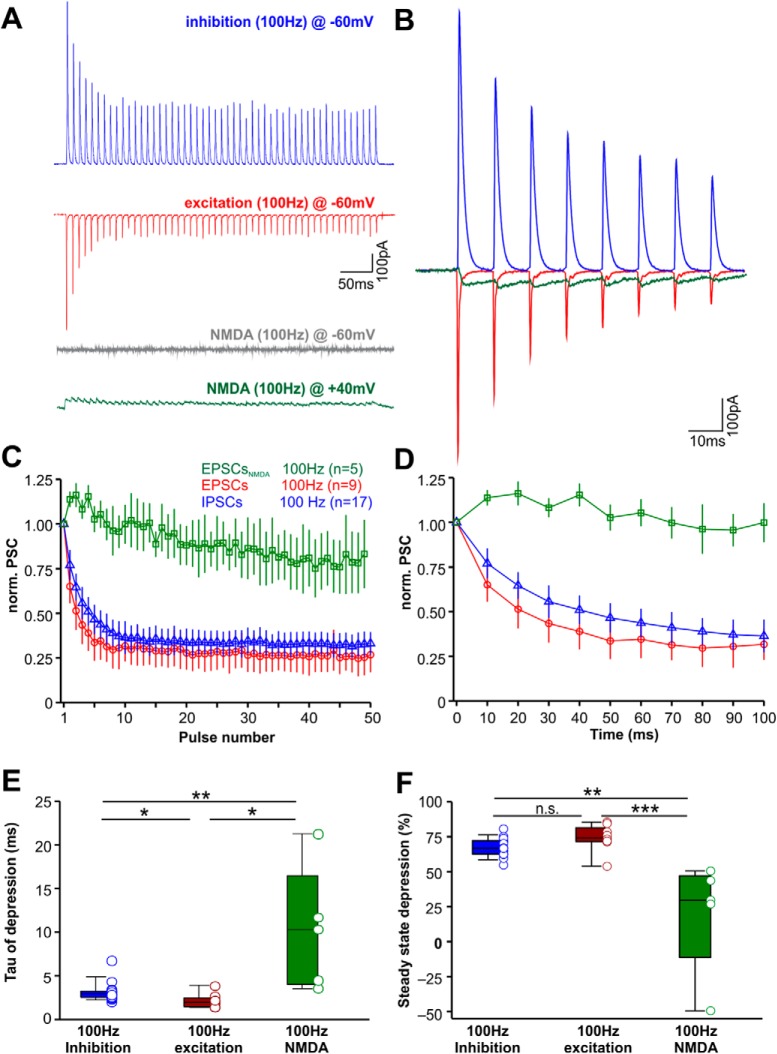
Short-term plasticity of excitatory and inhibitory inputs to SPN neurons. ***A***, Examples of inhibitory (blue), excitatory (red), and NMDAR (green)-mediated responses to 50 stimulations at 100 Hz (averages of 10 repetitions). Gray trace depicts the lacking NMDAR mediated response at –60 mV. ***B***, Overlaid and magnified first responses of the examples shown in ***A***. The NMDA trace (green) has been flipped to symbolize its excitatory nature. ***C***, Normalized and averaged current amplitudes to each of the 50 pulses of the 100-Hz train for NMDA currents (green), EPSCs (red), and IPSCs (blue). ***D***, First 100 ms of the plots shown in ***C***. ***E***, Time constant (τ) of the rate of depression acquired from fitting exponential decay functions to the functions shown in ***C***. ***F***, Summary of synaptic currents steady-state depression: 100% – (average current amplitudes in response to the last five pulses * 100). *p ≤ 0.05, **p ≤ 0.01, ***p ≤ 0.001, n.s. = non-significant.

To test for a balance between excitatory and inhibitory inputs in an adapted, more physiologic state, pharmacologically or electrically isolated inhibitory, excitatory and NMDAR-mediated synaptic currents were evoked in SPN neurons by applying 50-pulse fiber stimulation trains at 100 Hz (Fig. 7*A*). Since NMDAR-mediated currents can only be activated at depolarizing potentials positive to the EPSC reversal potential, they are shown as outward currents in the green trace in Figure 7*A*. For better visualization NMDA currents are flipped in the superimposed enlargement of all three current types for the first 100 ms of the response (Fig. 7*B*). Both fast inhibitory and excitatory currents showed pronounced short-term depression, calculated from a train of IPSCs and EPSCs, respectively, and normalized to the first peak (Fig. 7*C*,*D*). In contrast NMDAR-mediated currents showed very little depression (Fig. 7*C*,*D*). The time constant of depression was fastest for EPSCs (1.92 ms; 1.41/2.39 ms; *n* = 9), a little slower in IPSCs (2.88 ms; 2.53/3.23 ms; *n* = 17) and very slow in NMDAR-mediated currents (10.31 ms; 4.01/16.45 ms; *n* = 5; Kruskal–Wallis ANOVA on ranks followed by Dunn’s multiple comparisons; *p* ≤ 0.001; Fig. 7*E*). The average level of steady state depression was similar between EPSCs and IPCSs, but significantly lower in in NMDAR-mediated currents (inhibition: 66.70%; 62.57/72.05%; *n* = 17; excitation: 74.03%; 71.54/81.42%; *n* = 9; NMDA: 42.36%; 25.87/55.40%; *n* = 5; Kruskal–Wallis ANOVA on ranks followed by Dunn’s multiple comparisons; *p* ≤ 0.001; Fig. 7*F*).

Across the SPN, IPSCs were always larger than EPSCs. The position of each recorded neuron within the SPN was logged by taking a picture of the electrode position in 4× magnification. The SPN was then divided into 4 quadrants, which were correlated to the size of the currents. No correlation was found, suggesting a homogeneous distribution of excitatory inputs across the SPN with no distinct, spatially segregated subpopulations. The moderate, excitatory inputs to SPN neurons become visible as patterns of increased firing activity during sound presentation in about half of the offset-responding SPN neurons. The fact that these neurons have peristimulus as well as poststimulus responses raises new questions: do glutamatergic inputs, that drive peristimulus excitatory responses, interact with peristimulus inhibitory inputs that drive the poststimulus offset response, and if so, what is their impact on the offset response? To specifically address the role of NMDAR-mediated currents, compared to AMPAR-mediated currents, we fed the data acquired by voltage-clamp recording into a computational model to simulate both types of excitatory synapses and the inhibitory inputs to an SPN neuron.

### Computational modeling suggests that moderate and slow excitation affects offset-response latency

**Figure 8.**
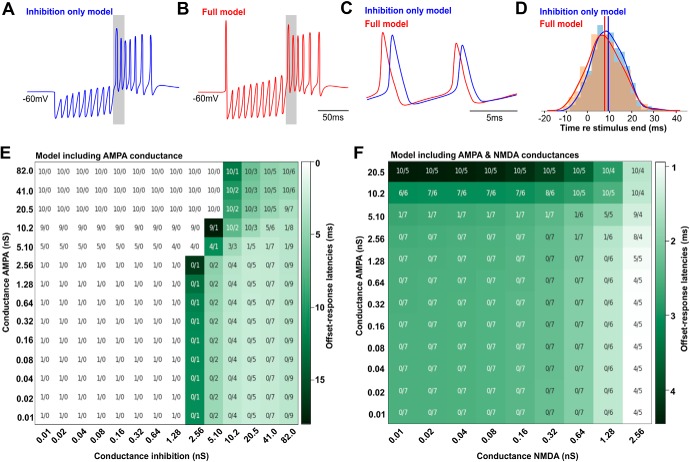
Excitatory input modulates offset-response timing in a computational model of SPN neurons. ***A***, Voltage trace of a SPN neuron’s response to a train of 10 stimuli using an inhibition only model shows 10 IPSPs followed by a burst of spikes at stimulus offset. ***B***, Voltage trace of a SPN neuron’s response to a train of 10 stimuli using the amended “full” model including AMPAR-mediated and NMDAR-mediated excitation shows a spike at stimulus onset followed by 10 IPSPs and then by a burst of spikes at stimulus offset. ***C***, Larger temporal resolution of the first two action potentials of the offset response (gray shaded area in ***A***, ***B***). ***D***, Distributions of offset-response latencies for the inhibition only model (blue) and the full model (red) for 500 repetitions when stochastic noise was added. The red and blue solid lines represent the mean offset latencies for the full and inhibition only model, respectively. ***E***, Heat map showing changes in offset-response latencies in relation to the strength of inhibition (*x*-axis) and AMPAR-mediated excitation (*y*-axis). The first number for each stimulus combination depicts the number of peristimulus spikes followed by the number of poststimulus spikes in the offset response. Darker green stands for longer latencies and lighter green for shorter latencies. ***F***, Heat map showing changes in offset-response latencies in relation to the strength of AMPAR-mediated responses (*y*-axis) and NMDAR-mediated responses (*x*-axis). Numbers and color code are the same as in ***E***. White areas in ***E*** specify stimulus combinations that do not generate offset responses.

A basic Hodgkin–Huxley model of SPN neuron firing was developed to test the effect of excitatory inputs of variable strength that are present in addition to inhibitory inputs. All synaptic conductances used for the following simulations were taken from the results presented in Figures 5–7. Conductance evoked by a single pulse, the synaptic depression during trains of synaptic stimulation and the duration of the stimulus train were taken into consideration while determining the range of conductances to be used in the model. Inhibitory conductances, recorded *in vitro* after a single pulse ranged from 11.5 to 80.5 nS with a median of 45 nS. We estimated the synaptic depression during a 100-Hz stimulation for 100 ms to be around 50%, resulting in a conductance of 41 nS (50% of 80.5 nS). For longer stimulation of 500 ms, a steady state depression estimated at 64%, results in minimal possible conductance values of 3.9 nS (64% of 11.5 nS). This is approximately the value where the inhibition becomes strong enough to generate a rebound spike in the model depicted in Figure 8*E*. Excitatory conductances were subjected to a similar approach, with minimum experimentally acquired excitatory conductances of 0.4 nS (minimal measured value with maximum depression) to 12.6 nS (maximal measured value with no depression). For NMDA currents, the range between the minimal measured value with maximum depression and maximum measured value with no depression was 0.3–1.9 nS. However, since NMDA currents depress only little and even initially facilitate (Fig. 7*C*,*D*), an average of 11% facilitation was added to the maximum measured value, providing an NMDA conductance of 2.1 nS. The values from *in vitro* experiments might be underestimating the synaptic conductances, as axons will be cut during the slicing procedure. To account for this caveat, we take advantage of the model to simulate a broader range of conductances. These physiologically feasible core conductances for inhibition, excitation and NMDA are covered in the matrices in Figure 8*E*,*F*. When using an inhibition-only model (Kopp-Scheinpflug et al., 2011) with adapting inputs, SPN neurons reliably fired a burst of offset responses following a 100-ms train of IPSCs presented at 100 Hz (Fig. 8*A*). The original model was amended in the full model by adding adapting AMPAR-mediated and NMDAR-mediated currents (Fig. 8*B*). This model, now including inhibition and excitation, caused neurons to fire spikes not only at the end of the stimulus train but also at the onset of stimulation (Fig. 8*B*). Comparing the offset responses elicited by either model revealed that the model incorporating excitation (Fig. 8*C*, full model) generated shorter offset-response latencies. Adding stochastic noise to either model introduced a jitter to the offset-response latencies (Fig. 8*D*). However, the average latencies for the full model (7.55 ± 0.36 ms) were still shorter compared to the inhibition only model (9.12 ± 0.33 ms) by 1.57 ms (two-tailed *t* test: *p* = 0.0012; *t* = 3.245; *DF*: 998; Fig. 8*D*, red and blue vertical lines).

To assess how the balance between excitation and inhibition affects offset-response latencies, both conductances were independently varied in strength and the corresponding changes in latencies shown in a heat map (Fig. 8*E*; model including AMPA currents). Specific combinations elicited both onset and offset responses and were thus most comparable to the ON-OFF type neuronal responses *in vivo*, while in most cases no onset response was generated. Comparing the offset-response latencies for vertical columns of a specific inhibitory conductance (e.g., 41 nS in Fig. 8*E*) and increasing AMPA conductances revealed that varying the AMPA conductance alone mediates the spike at stimulus onset, but did not significantly shorten the latencies of spikes in the offset-response (Fig. 8*E*). On the contrary, increasing the AMPA response beyond the physiologic estimates will prolong the offset-response latencies due to the occurrence of peristimulus spikes that interfere with the refractory period of the offset-response spikes. The shortest offset-response latencies were obtained with a combination of strong inhibition (41 nS) coupled with both AMPA and NMDA conductances (Fig. 8*F*). In the latter condition, varying the AMPA conductance between 0 and 2.56 nS (*y*-axis) did not change the offset-response latency. However, increasing the NMDA conductance (*x*-axis) for any of these AMPA conductances increasingly shortened the offset-response latency. Increasing the AMPA conductance further (≥5.1 nS), caused continuous peristimulus spike firing which then prolonged offset-response latencies due to refractory interactions. A combination of inhibition and NMDA conductance alone did not have an effect on offset-response latencies (data not shown).

These simulations suggest that although offset responses can be generated by exclusively activating the chloride conductance, the slow NMDA conductance that accompanies fast AMPA-mediated responses serve to accelerate the offset response. Overall, a longer EPSC decay time results in a shorter latency, as the effect of the excitation is carried forward into the rebound, providing additional excitatory drive. We found that the shortest offset-response latency was generated with simulated EPSC decay time constants of 30 ms, but even EPSC decay times of 10 ms accelerate the offset response.

### Simultaneous activation of excitation and inhibition *in vitro* results in a reduced net hyperpolarization of PSPs and shorter offset-response latencies

**Figure 9.**
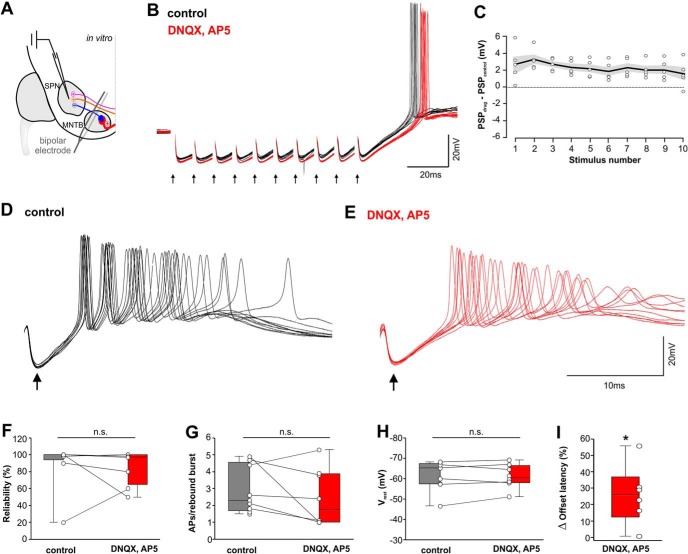
Excitation improves offset-response timing. ***A***, Schematic sound-offset circuit depicting the position of the fork-electrode for simultaneously stimulating excitation and inhibition. ***B***, 100-Hz synaptic stimulation (black arrows) elicited an offset response in control (black) and during blockade of excitation (red). Stimulus artifacts are removed for clarity. Postsynaptic potentials were more depolarized in control (black). ***C***, Average difference in amplitudes of PSPs (not spikes) between blockade of excitation and control was plotted against stimulus number within each train (circles are averages of 10 trials/cell; *n* = 5 cells). Black line and gray-shaded area represent mean ± SEM. ***D***, ***E***, Higher temporal resolution of the offset responses of the cell shown before (***D***) and after (***E***) the blockade of excitation. ***F***, Reliability of offset responses in 10 consecutive traces (as shown in ***D***, ***E***; 100%: at least one rebound spike/trace); *n* = 9. ***G***, Average number of rebound spikes (10 trials; *n* = 9). ***H***, Resting membrane potential averaged over 10 ms before the start of synaptic stimulations (*n* = 9). ***I***, Normalized increase in offset-response latencies after blocking excitation (*n* = 6). *p ≤ 0.05, n.s. = non-significant.

Excitatory inputs to SPN neurons are not a prerequisite for generating offset responses (Kopp-Scheinpflug et al., 2011). However, computational modeling (Fig. 8) suggests that excitation might shape the temporal precision of the offset responses. Here we compared synaptically evoked offset responses in SPN neurons in control condition and during blockade of excitatory inputs by simultaneously stimulating both the MNTB and IAS using a fork-like bipolar stimulating electrode (Fig. 9*A*). At the end of a 100 ms, 100-Hz train of stimuli, SPN neurons reliably generated an offset response consisting of a burst of spikes (Fig. 9*B*). The blockade of glutamatergic transmission during 100-Hz stimulation for 100 ms (Fig. 9*B*, red trace) caused a hyperpolarizing drop in the net amplitude of the evoked PSPs (Fig. 9*B*). In control condition, the stimulation of synaptic input triggered a combined response of excitation and inhibition resulting in a net hyperpolarization of –80.9 ± 0.3 mV (*n* = 5) averaged over the stimulus train. However, blocking excitation caused the PSPs to drop by 2.6 ± 0.1 mV (*n* = 5) toward more hyperpolarizing voltages (Fig. 9*B*,*C*). The voltage difference between drug and control condition revealed a small, yet sustained depolarizing drive over the whole stimulus time (Fig. 9*C*), consistent with our simulations which predicted the presence of a small, slow excitatory conductance which extends into the temporal window of the offset response.

Blockade of excitatory inputs (only the last IPSPs of the trains shown in Fig. 9*D*,*E*) did not significantly change the reliability of generating an offset response: in 10 consecutive input trains, offset bursts were generated in 90 ± 9% of trains in controls and 86 ± 7% of trains during blockade of excitation (Mann–Whitney rank-sum test: *p* = 0.551; Fig. 9*F*). The number of spikes within each burst of the offset response was also not significantly different between the control condition (2.30; 1.7/4.6; *n* = 9) and following the blockade of excitation (1.75; 1.0/3.8; *n* = 8; Mann–Whitney rank-sum test: *p* = 0.359; Fig. 9*G*). To probe whether tonically active excitation is present in the SPN, we compared the resting membrane potential in absence of synaptic stimulation before and during blockade of excitation, but no significant difference was found (V_rest_control: –61.8 ± 2.4 mV; V_rest_drug: –61.1 ± 2.0 mV; two-tailed *t* test: *p* = 0.832; *t* = 0.215; df = 15; Fig. 9*H*). Functionally, the blockade of excitation caused longer offset-response latencies. Depending on the exact positioning of the stimulating electrode and patch pipette in each *in vitro* preparation, the latencies of synaptically-evoked offset-responses varied between cells from 5.6 to 68.5 ms (control) and from 7.2 to 84.2 ms (DNQX/AP5). However, each paired recording showed an increase in latency during blockade of excitation and resulted in a significant increase of 30.68 ± 6.71% in offset-response latency when excitation was blocked (two-tailed paired *t* test: *p* = 0.01; *t* = –4.571; df = 4; Fig. 9*I*).

In conclusion, our data show that the right balance of moderate, slow excitation and strong inhibition will accelerate acoustically-evoked offset responses. Besides this faster offset firing, the presence of additional excitation also significantly reduces intensity-dependent changes in response latency. Based on the distributions of synaptic inputs within the SPN, similar CFs, thresholds and spontaneous rates between neurons with ON-OFF type and OFF-only type responses, we conclude that the different response patterns are not arising from two different types of neurons but rather reflect differences in the balance of excitation and inhibition.

## Discussion

SPN neurons are reliable detectors of sound offsets and respond with a burst of spikes time-locked to the end of the stimulus. The latencies of these offset responses are short and level-invariant over a large range of supra-threshold sound intensities, which are prerequisites for sound-duration encoding and gap-detection (Forrest and Green, 1987; Brand et al., 2000; Faure et al., 2003; Pérez-González et al., 2006). For half of the neurons with offset responses, additional excitatory inputs were observed in *in vivo* recordings. These neurons exhibited even shorter offset-response latencies and stronger level invariance of less than 10 µs/dB. Pharmacological manipulation and computational modeling showed that while inhibition alone can reliably trigger a post-inhibitory rebound response at stimulus offset; excitation alone will not generate an offset response. However, the presence of slow, NMDAR-mediated excitation facilitates the rebound depolarization and speeds up the latencies of offset responses.

### Why don’t all SPN offset cells show peristimulus excitation?

Shorter latencies and greater resistance to level dependent latency shifts due to additional excitation provide advantages for computation of sound duration and for detection of silent gaps in noise. So why do not all SPN neurons with offset responses benefit from this advantage? Our immunocytochemical data (Fig. 1) show uniform distributions of excitatory and inhibitory inputs throughout the SPN, suggesting that the observed differences in the strength of excitation and inhibition between ON-OFF type and OFF-only type neurons may not manifest in different synaptic input patterns. This is in agreement with the lack of differences in CFs, spontaneous rates or thresholds between ON-OFF type and OFF-only type neurons. Whether or not neurons in the SPN could be classified into distinct subpopulation has been discussed for 40 years. Ollo and Schwartz (1979) used Golgi impregnations to assess the morphology of mouse SPN neurons. They described slight morphologic differences such as triangular, elongated or polygonal shapes, but stated that these were “not sufficiently distinct to warrant division into different cell types.” More complex approaches combined the morphologic description with either immunostaining for the neurotransmitter used by SPN neurons (Helfert et al., 1989) or with neural tracing experiments (Schofield, 1991). As a result five SPN cell types that project to the ipsilateral inferior colliculus (IC) were described: (1) large round glycinergic neurons, (2) large round GABAergic neurons, (3) small, round, projecting bilaterally to IC glycine-negative neurons, (4) small neurons with only ipsilateral IC projections, (5) small neurons with ipsilateral IC and contralateral cochlear nucleus projections (Helfert et al., 1989; Schofield, 1991). Another approach to classify SPN neurons was taken by Felix and co-authors, who reported only subtle differences in the intrinsic properties of SPN neurons and suggested that these might be caused by gradients of potassium currents (Felix et al., 2013). Whether or not the bursting cells in the dorsolateral SPN region can form a particular subtype or are still subject to developmental change is not yet clear. In conclusion, the occurrence of ON-OFF type responses in a subset of SPN offset cells could be the result of differences in the balance of existing excitatory and inhibitory inputs. Previous studies have shown that the assessment of the excitatory-inhibitory balance might be confounded through the use of anesthetics which either block NMDA currents (ketamine-based anesthesia) or alter inhibition (barbiturate based anesthesia) (Steinert et al., 2008; Felix et al., 2012). Here, we used a fentanyl-based anesthesia, which binds µ-opioid receptors and therefore should not directly interfere with either excitation or inhibition in the auditory brainstem.

We conclude that the occurrence of ON-OFF type and OFF-only type neurons in SPN is neither a developmental nor an experimental (anesthesia-based) effect. Instead, ON-OFF or OFF-only responses in SPN neurons are likely caused by differences in the strength of excitation and inhibition whose activity- or context-dependent control will have to be investigated to further our knowledge on encoding sound offsets.

### Facilitating the post-inhibitory rebound

The excitatory input alone does not generate offset action potentials, yet it is sufficient to modify the offset responses generated via a post-inhibitory rebound mechanism (Felix et al., 2011; Kopp-Scheinpflug et al., 2011). A subthreshold inhibitory input aiding a subthreshold excitatory input to increase temporal precision is generally referred to as post-inhibitory facilitation and has been shown in other SOC neuron types (Dodla et al., 2006; Beiderbeck et al., 2018). This is not the case in our present SPN data, since here the inhibitory input on its own is sufficient to generate offset spikes. However, the slower time course of the additional excitation mediated via NMDARs allows it to extend its depolarization into the temporal window of the post-inhibitory rebound just enough to accelerate the offset response. Such an extension of excitation into the post-inhibitory rebound would require sustained excitatory responses throughout the duration of the stimulus. The fact that the peristimulus excitatory responses observed in the ON-OFF type neurons *in vivo* occur as either sustained (38%) or onset (62%) responses is likely due to most of the excitatory inputs being subthreshold caused by dominant inhibition during sound presentation, rendering subthreshold EPSPs invisible to our single cell extracellular assessment of spiking. Such subthreshold, peristimulus excitation was demonstrated by pharmacological blockade of inhibition *in vivo* (Kulesza et al., 2007). Based on these previous findings and our present results, it seems likely that, at least part of the peristimulus excitatory inputs are subthreshold and may serve modulatory functions rather than to form a reliable representation of sound onset. This would be in agreement with the suggestion that sound onsets and offsets are encoded in segregated pathways within the auditory brain (Scholl et al., 2010; Anderson and Linden, 2016; Kopp-Scheinpflug et al., 2018; Sollini et al., 2018). While the SPN strongly qualifies for encoding offsets, sound onset information is likely provided by different neuronal pathways such as for example the ventral nucleus of the lateral lemniscus.

The behaviorally relevant readout in the present data set is the reduction in offset-response latencies, which can decrease gap-detection thresholds (Yassin et al., 2014). The slow, lasting excitation responsible for the faster offsets, could represent the underlying cellular equivalent to adding background noise in a behavioral gap detection task, where gap detection thresholds have been significantly decreased (better), compared with a condition when no background noise was provided (Horwitz et al., 2011).

However, faster is not always better, especially when temporal precision (as measured by jitter) is equally good. An alternative interpretation is that the modulation of offset-response latencies might present a tool to accelerate or delay the time point of the offset response depending on the balance between excitation and inhibition. Such a shift in latency could present a homeostatic adaptation to a changing balance between excitation and inhibition as might occur during aging or following acoustic trauma.

### Reducing the level dependency of the offset response

The offset-response latency depends on sufficient hyperpolarization and acceleration of the membrane time constant via recruiting additional ionic conductances such as I_H_ (Kopp-Scheinpflug et al., 2011). Hyperpolarization of the membrane activates I_H_, which accelerates of the membrane time constant and shortens the offset-response latency. If, however, I_H_ is already maximally activated by hyperpolarization of the membrane, any further hyperpolarization might prolong the offset-response latency as it takes the membrane longer to move from –100 mV (SPN IPSP reversal potential) to + 40 mV (sodium channel activation voltage) than for example from –80 to +40 mV. This is likely what happens with the trend of increasing offset-response latencies with increasing intensity seen in Figure 3*A*,*B*. At higher sound intensities, multiple MNTB axons will be recruited and due to input summation, the net-depression of inhibitory inputs in SPN neurons will be reduced, resulting in strong hyperpolarization of the membrane voltage even at the end of the stimulus train. The recruitment of additional excitatory conductances as observed in the present study only at higher stimulus intensities is likely to have a similar effect as I_H_, in shunting the inhibition, providing a depolarizing drive and shortening the offset-response latencies.

In contrast, at lower sound intensities only few MNTB axons might be recruited to inhibit SPN neurons. These inhibitory inputs will depress over time as shown for the SPN in this study or for the LSO and MSO in other studies (Couchman et al., 2010; Walcher et al., 2011; Roberts et al., 2014) and an offset response is generated at the time point when the intrinsic depolarizing drive dominates the hyperpolarization. In the experimental *in vitro* condition, especially for long stimulations of several hundred milliseconds, this can lead to rebound responses even before the end of the stimulus train (unpublished observations). *In vivo*, such rebound response before the end of a sound was never observed, suggesting that *in vivo* the depression of collective inhibitory inputs is low and the hyperpolarization at the stimulus end is strong.

### Implications of level-invariant offset responses for gap-detection and sound-duration encoding

The threshold for detecting brief silent gaps in noise provides a valuable analytical tool to measure temporal resolution in auditory processing (Moore, 1997). Gaps as short as 2–3 ms can be detected by humans (Penner, 1977) and rodents (Ison, 1982) and it has been suggested that to detect a 3-ms gap, auditory neurons need to encode onsets and offsets of sounds with a temporal acuity of ∼1 ms (Oertel et al., 2017). Gap-in-noise stimuli have also proven very helpful in determining the ability of the auditory system to encode sound offsets as a parameter independent of sound onsets (Pratt et al., 2005).

Behaviorally, sound offsets are an important cue for sound-duration encoding. Neurons in the auditory midbrain that are sensitive to sound duration act as coincidence detectors that only fire action potentials if excitatory postsynaptic responses evoked by the onset of sound temporally coincide with excitatory postsynaptic responses evoked by the offset of sound (Casseday et al., 1994; Aubie et al., 2009, 2012, 2014; Sayegh et al., 2011; ). Duration sensitive neurons have been classified according to their ability to preferably encode sounds of different durations and are referred to as short-pass, bandpass and long-pass duration tuned neurons. According to the current models of sound-duration encoding, offset excitation is needed in the coincidence detection bandpass mechanism but not in the anti-coincidence detection short-pass mechanism (Aubie et al., 2012). This is in agreement with previous results showing that temporally precise SPN offset responses vary with stimulus duration and provide an inhibitory projection to the auditory midbrain (Kadner et al., 2006; Kopp-Scheinpflug et al., 2011) where they are suggested to generate a post-inhibitory rebound excitation (Pollak et al., 2011). Interestingly, both the detection of gaps (Moore, 1997) and the discrimination of different sound durations (Klink and Klump, 2004) are relatively independent of changes in suprathreshold sound level. Especially the duration discrimination of longer sounds (>50 ms) has been reported to not show an intensity effect (Henry, 1948). The level-independence and short latency of SPN offset responses over an extremely large range of intensities as shown in the present study provide a perfect function for the ON-OFF type SPN neurons in the encoding of longer sound durations. Deficits in SPN offset encoding might therefore result in difficulties in processing sound offsets in downstream auditory areas like the IC or the MGB (Anderson and Linden, 2016).

## Synthesis

Reviewing Editor: Leonard Maler, University of Ottawa Decisions are customarily a result of the Reviewing Editor and the peer reviewers coming together and discussing their recommendations until a consensus is reached. When revisions are invited, a fact-based synthesis statement explaining their decision and outlining what is needed to prepare a revision will be listed below. The following reviewer(s) agreed to reveal their identity: Christian Sumner. Note: If this manuscript was transferred from JNeurosci and a decision was made to accept the manuscript without peer review, a brief statement to this effect will instead be what is listed below.

Editorial comments. The revised Ms has now been re-reviewed by two reviewers. One reviewer (#1) had previously seen the Ms submitted to JNS, while a second reviewer (#2) was reviewing the Ms for the first time. Reviewer #1 found that the authors had made most of the revisions that he/she had requested. This reviewer still has some fairly minor concerns. If you can address these concerns and, in your rebuttal and redlined copy, clearly indicate the revisions, then I will not request a re-review by Reviewer #1; I will simply check to make sure your revisions have addressed all their concerns.

Reviewer #2, seeing this Ms for the first time, found that it presented interesting and detailed work, was mostly well written and provided important analyses of the “relationship between NMDAR activation and latency of post-inhibitory responses that has intuitive relevance for sound offset detection”. However, this reviewer did find many points that need addressing before this Ms is suitable for eNeuro.

I will request that this reviewer re-review your next iteration and would hope that you can take care of the points raised in her/his careful review.

Again, please make sure that you have a clear rebuttal and both a redlined and 'clean' version of your Ms.

Dr. Leonard Maler

Reviewer #1

Line 200: “SPN neurons receive glycinergic and VGLUT2 dominated glutamatergic synapses”. Can you really claim dominated here? The text says stronger than VGLUT1. The example in figure 1 certainly looks stronger, but for a title of dominated it would be nice to see further quantification, and more than one example. Can they summarise data across animals in some way? And perhaps change the title if quantification suggests less than domination of one type. 

Line 211: “ and rather somatic than in...” -&gt; “and somatic rather than in....” 

Figure 2: It wouldn't hurt to label the response type examples in C,D,E (on-off, off, “other” or suitable label). 

L285. I don't know why they added this comma. Better without. 

L304. “at that rate.” I don't understand what it means to “tonically suppress at that rate”. It seems to imply that the reduction in firing rate due to the inhibition is precisely that of the spontaneous rate of a single incoming neuron. Is that right? Either delete the phrase or be more specific. 

L344: “suggesting that it is not a by tens of milliseconds delayed excitatory input” Sentence requires attention. 

L328: “Excitation in SPN neurons is effective only at higher sound intensities.” It is not obvious to me why the text that follows provides evidence of this statement. “Peristimulus excitatory thresholds area higher than poststimulatory thresholds” seem more apt. 

L353-356. Split this sentence up. 

L452: deleting “for the offset response using” leads to an ungrammatical sentence. 

L454: The distribution of offset latencies in Figure 8D look very similar for the full model and inhibition model. I'm not sure it supports their subtitle “Computational modeling suggests that moderate and slow excitation affects offset-response latency”. They should quantify this difference. I can see that there is a consistent trend in latency in the parametric variation in E and F, but it looks small relative to the variance of a stochastic model. 

Figure 8. It would make more sense for the vertical axis in 8F to represent AMPA, to match 8E. 

L373: “the slow NMDA conductance that accompanies fast AMPA-mediated 472 onset responses serve to accelerate the offset response”. This does not appear to be well supported by the modelling. First the effect of varying AMPA conductance is quite non-monotonic. Secondly, it appears to be quite subtle in the range of most of AMPA conductances on the Figure 6. It looks like the effect of the AMPA is closer to 1ms in the physiological range of the NMDA (mean is &lt;1 nS). I wonder if the narrative might be better if they reversed the last two sections? Presenting the data on latency before the models.

Reviewer #2

Major/general comments: 

1. While the developmental angle may have been removed, the age ranges used for remaining experiments causes some concern since the three methods used, IHC (P21-35), in vivo (P42-112), and in vitro (P15-22), were all done at practically non-overlapping ages. Of these groups the in vitro physiology is most worrisome since it is known that NMDAR or subunit expression can change from this developmental time to when the in vivo work takes place and the NMDAR responses form a major component of the modeling work to explain the in vivo findings. If there is good evidence for lack of changes in this regard available they should be brought to the fore. Basic intrinsic properties like input resistance and resting potential should be provided for these cells for comparison with other studies. 

2. Given modern digital quantification methods, if the results from the immuno data are going to be discussed in terms of relative expression levels and location and leaned on in the discussion to make conclusions (L569) some effort should be made at quantification since only a few example images are provided. How was tonotopic assessment made and were sufficient replications made to power such an analysis? Additionally, if relative vGlut type is going to be considered it should be in the same tissue sections. Are there other transporter expression studies that can be of use in this regard? While it is recognized that the immuno data could be considered qualitative confirmation for physiological results, it is potentially misleading to the field to draw conclusions without some analysis.

Minor/specific comments: 

1. L74,74: it is not clear what is being indicated and a reference seems to be needed. 

2. L93: this mention of gap detection needs referencing. 

3. L141: below should be above. 

4. L157: coma after the 1. 

5. Figure 1D,G: should the nuclei be labeled like in A for clarity as to the only thing changing is VGlut type? 

6. Figure 2: recommend labeling the response type at the top of C-E. 

7. Figure 2H: is the rate an average for the whole peri/post period? Should be noted in legend. 

8. Figure 2C-E: why so loud at 80 dB if testing at CF? Were responses similar at 20 dB above threshold? 

9. Figure 2C,G: is the primary-like ON responses what would be expected from what is known about the firing properties of octopus cells? Onset only would seem more expected as I recall, regardless it should be discussed. 

10. Figure 2H: half of the ON-OFF cells fire more during the stimulus than after and appear to separate out into two groups. This would seem to merit another example trace set to contrast with C. Related to comment 6 above, are the low ones more onset only responses and the high ones more sustained? 

11. Figure 2F: is the Y axis really the “Onset” or is the average of the ON/OFF period being used. If onset, what part of the response period was used. 

12. Figure 3A,B: what each dot consists of is not provided in the legend. 

13. Figure 3A: the firing during the sound presentation seems very low and not terribly different from B except that there seems to be an onset response that appears at higher intensities. Is this cell also shown in the Fig. 2 PSTH's? If so note it. Best to give each cell a number when relying on representative data so reader can easily compare across data and include the CF of example cells. 

14. Figure 3C,D: what intensity level/levels was used for these results? Should be noted in legend. 

15. L285-290: It is not clear what the logic is for CF being connected to “differently balanced excitatory vs. inhibitory inputs.” 

16. L295: While there was no significant difference in CF's, is there really sufficient numbers of data points to support this conclusion? 

17. Are voltages corrected for liquid junction potential? 

18. L370: Relative strength of E/I should be discussed in terms of driving force. Conductance calculation method should be noted. 

19. L393,397 still have “mature” which might not be appropriate for tissue as young as P15 and seems to be a hold-over from the developmental story.

20. L397: “quite small” needs to be qualified or referenced. NMDAR currents in LSO and MSO are similar. Also, how does this small size relate to the ability to assist with post-inhibitory depolarization? Why were voltage-clamp recordings not used to estimate the size of Ih currents after hyperpolarization for comparison? 

21. L406,407: the statement that NMDAR currents could only be measured at positive potentials is contradicted by the presented results and the rationale for its relevance to the following statement is unclear. 

22. L430,431: Since no analysis is presented for tonotopic differences how was this statement determined? I one does not look for something then they will not find it. The authors have not demonstrated how they looked for tonotopic difference for any of the three data sets. 

23. Figure 8F: what is the inhibitory conductance level used? 

24. Figure 8F: it is not entirely clear whether conductances from previous figures can be directly compared to those shown in 8F, but it so the average AMPA conductance from 5E is ~70 nS and would be at the extreme end of 8F and the NMDA conductance in 6F average is ~0.9 nS so somewhere near the middle of 8F range but there were rarely cells at the 2.56 nS level where the quick transition was noted. Regardless there should be some discussion of relevance of in vitro findings to model performance. 

25. L566,567: rationale for advantages needs to be added and references. 

26. L589-597: consider whether this paragraph is needed. At least scrub the part about no developmental changes in the data set since no data is presented. 

27. L623-626: I does not seem appropriate to say that peristimulus is largely subthreshold since some cells exhibited robust onset firing in vivo and some even had more peristimulus firing than post in Fig. 2H. 

28. L629: second “offset” should be an onset? 

29. L701: typo, neuropile. 

30. L804: “orange” should be red 
